# Exploring Impacts of Taxes and Hospitality Bans on Cigarette Prices and Smoking Prevalence Using a Large Dataset of Cigarette Prices at Stores 2001–2011, USA

**DOI:** 10.3390/ijerph14030318

**Published:** 2017-03-20

**Authors:** Lance S. Ballester, Amy H. Auchincloss, Lucy F. Robinson, Stephanie L. Mayne

**Affiliations:** Department of Epidemiology and Biostatistics, School of Public Health, Drexel University, Philadelphia, PA 19104, USA; lanceballester@gmail.com (L.S.B.); lucy.f.robinson@drexel.edu (L.F.R.); stephanie.lynn.mayne@drexel.edu (S.L.M.)

**Keywords:** places, smoking, environment, tobacco control, tobacco price, smoking cessation policies, secondhand smoke

## Abstract

In the USA, little is known about local variation in retail cigarette prices; price variation explained by taxes, bans, and area-level socio-demographics, and whether taxes and hospitality bans have synergistic effects on smoking prevalence. Cigarette prices 2001–2011 from chain supermarkets and drug stores (*n* = 2973) were linked to state taxes (*n* = 41), state and county bar/restaurant smoking bans, and census block group socio-demographics. Hierarchical models explored effects of taxes and bans on retail cigarette prices as well as county smoking prevalence (daily, non-daily). There was wide variation in store-level cigarette prices in part due to differences in state excise taxes. Excise taxes were only partially passed onto consumers (after adjustment, $1 tax associated with $0.90 increase in price, *p* < 0.0001) and the pass-through was slightly higher in areas that had bans but did not differ by area-level socio-demographics. Bans were associated with a slight increase in cigarette price (after adjustment, $0.09 per-pack, *p* < 0.0001). Taxes and bans were associated with reduction in smoking prevalence and taxes had a stronger association when combined with bans, suggesting a synergistic effect. Given wide variation in store-level prices, and uneven state/county implementation of taxes and bans, more federal policies should be considered.

## 1. Introduction

In the U.S., smoking prevalence has declined over time, from approximately 33% in 1980, 26% in 1990, 23% in 2010, and 18% in 2013 [1]. Policies that aim to generate revenue and reduce smoking have likely contributed to the decline [2,3]. Nevertheless, cigarette smoking continues to be a major public health problem in the U.S., resulting in approximately 480,000 deaths each year from smoking-related diseases [3].

Work to date suggests that there is considerable heterogeneity in cigarette tax policies between and within states; and suggests that heterogeneity in pricing may be hampering public health tobacco control efforts. Yet, only a few U.S. studies have provided a detailed characterization of cigarette price heterogeneity within states and counties by examining store-level prices. Three U.S. studies analyzed store-level price data [4,5,6] but they all focused on short time periods (≤2 year) and two focused on only a single geographic region [4,6].

One potential reason for local heterogeneity is due to differences in how much tobacco taxes are passed onto consumers. In the U.S., tobacco tax policy has used indirect taxes levied on the tobacco producer or vendor (“excise taxes”). Results of prior studies have been mixed regarding whether tobacco excise taxes are in fact fully passed on to the consumer [7,8], only partially passed on [5], or over-shifted [4,6,9,10,11]. Several studies have identified geographic differences in the pass-through rate [4,5] and two studies found differences by socioeconomic status [5,6], factors which may contribute to local variation in cigarette prices. 

The implementation of smoking bans is another potential contributor to local variability in cigarette prices. If bans decrease demand, then manufacturers may decide to lower prices and reduce their profit margin. Alternately, manufacturers may decide to accept lower volume sales and raise prices to maintain revenue. To date, the relationship between bans and prices is understudied. Only one study addressed this question using state-level price data from 1960–1990, and found significantly lower prices in localities with more stringent anti-smoking laws [9].

In general, previous studies have described declining smoking prevalence following the introduction of tobacco tax increases [2,12] and indoor smoking bans in workplaces [13]. Recently, a few studies have analyzed effects of smoking bans in a small number of localities (including hospitality bar and restaurants) and also found them associated with reductions in smoking prevalence [14,15,16,17]. However, no studies have evaluated whether cigarette taxes and smoking bans have a synergistic effect on smoking prevalence.

In sum, no study to date has used store-level price data to evaluate associations between hospitality bar and restaurant bans on cigarette prices including a large geographic area over a long period of time. To address this knowledge gap, this study used a large U.S. cigarette price dataset from 2001 to 2011 to document variation in store-level cigarette prices and associations with federal and state cigarette taxes, hospitality bans and area-level socio-demographic characteristics. In addition, we tested whether excise taxes were uniformly passed to consumers irrespective of area-level socio-demographics. We then explored ecologic variation in smoking prevalence by state and county and the contribution of store-level cigarette prices, state and federal cigarette taxes, and hospitality bans to smoking prevalence; and tested whether the presence of hospitality bans changes the impact of cigarette taxes on smoking prevalence.

## 2. Materials and Methods

### 2.1. Data

#### 2.1.1. Cigarette Prices

Pricing data 2001–2011 came from Information Resources Inc.’s (IRI) Academic Dataset, a panel of large chain supermarkets and drug stores in 47 U.S. market regions located in 41 states [18]. The current study includes 3084 large chain outlets (from 128 and 13 large chain grocery and drug store companies, respectively) located in 483 counties. Examples of the chain companies are: Albertson’s, A&P, Food Emporium, Pathmark, Walgreens, CVS, and Rite Aid [19].

Cigarette prices were available for all Universal Product Codes sold at a store. In order to reduce price variation simply due to varying cigarette size and package size, we restricted the analytic sample to the most popular package size and type of cigarette in the dataset: king-sized and long-sized cigarettes (99% of all cigarette revenue) and single packs (57% of all cigarettes revenue). Cigarette price reflects the “shelf price” and includes excise taxes that the retailer may pass to the customer, store-level promotions and retailer coupons, but does not include sales tax and manufacturer coupons. 

There was no objective way to classify brands thus we included all types and did not differentiate (premium, standard, generic); note that upwards of 70% of tobacco sales come from premium or standard brands [20,21].

#### 2.1.2. Cigarette Taxes

State and Federal cigarette taxes for every month of the study period came from the Tax Burden on Tobacco [22]. Because cigarette taxes are applied as excise taxes, the tax may or may not be incorporated into the “shelf price” (at the discretion of the seller). County/municipal cigarette taxes were not used because there was no reliable source for historic data 2001–2011. Data that we manually collected from state websites suggested that the county/municipal taxes were not widespread and were small. For example, available data from 2011 indicated only 13.5% of stores in our dataset were in areas with county/municipal taxes; tax median $0.60 (range 0.05–0.85). The exception to this was New York City and Chicago, where local taxes were very large (1.50 and 2.68, respectively), thus, stores (*n* = 111) in these counties (*n* = 6) were excluded (Note that Alaska was not in our dataset). After this exclusion, there were 2973 stores in 477 counties for analyses.

#### 2.1.3. Smoking Bans

State and county workplace smoking bans and date of enactment came from the American Nonsmokers’ Rights Foundation (2001–2011) [23]. A county within a state that adopted a ban was also considered to have adopted the ban [24].

The present study only included timing of hospitality indoor smoking bans requiring that *all* restaurants and free-standing bars be 100% smoke-free. We excluded: (a) restaurant-only or bar-only bans (maximum of only 4% of counties in our dataset in any year); (b) non-hospitality workplace smoking ban policies (in the U.S., most establishments had voluntarily banned indoor smoking by the late 1990s, thus, before the study period and years before the government enacted smoking bans in non-hospitality establishments [25]).

#### 2.1.4. Smoking Prevalence

Estimated county smoking prevalence for each year came from Dwyer-Lindgren et al. (2014) [26]. Their estimates were derived from the Behavioral Risk Factor Surveillance System (BRFSS) self-reported data on adult smokers [27]. BRFSS does not provide county-level estimates for much of the U.S., thus, Dwyer-Lindgren et al. predicted annual smoking prevalence (daily and any smoking) via a validated small area estimation method that utilized BRFSS, census demographics, state cigarette sales data, and other data (see their publication for more details [26]). We derived non-daily smoking prevalence by subtracting daily smoking prevalence from total smoking prevalence. For models where smoking was an outcome variable, all variables were summarized at the county-level.

#### 2.1.5. Temporal Aggregation

Data were summarized into annual measures. Adapting methods developed by others [7], at each store location, dollar and unit sales were first aggregated from weeks to years and from Universal Product Codes to standardized brand name to formulate an average yearly price per pack (PPP) for each brand and each store location. These yearly brand-store PPP were then used to create a weighted average price, where weights are based on the proportion of each brand sold during the entire study period. Thus, the weights resulted in yearly PPP independent of temporal brand buying patterns which could have over- or under-estimated cigarette price (prior work has found that some smokers respond to tax increases by switching to more expensive products or by buying less expensive brands, respectively [28]).

Because some tobacco regulations are implemented mid-year, annual cigarette state tax at each store was weighted by proportion of packs sold before mid-year and after mid-year: Sum (Weekly Tax × Weekly Packs Sold)/Yearly Packs Sold. In the same manner, bans were calculated to account for mid-year policy changes. All prices and taxes were adjusted to 2010 dollars based on the U.S. Bureau of Labor and Statistics Consumer price index [29].

#### 2.1.6. Census Data and Linking Stores to Census Data

Census data come from the middle of the study period: the American Community Survey (ACS) 5-year summary file 2005–2009 [30]. Each store was assigned to the population-weighted centroid of its block group (*n* = 2822) and block groups within 1-mile of each store were selected to represent characteristics of residents around each store (This approach was used because many chain stores were located in non-residential areas). Thus, census data in our analyses consist of average socio-demographics of the block groups surrounding each store. Census data were used to construct area-level variables related to age, race, and socio-economic status; these were included due to their potential spatial patterning (thus could confound the tax-price association) as well as their associations with smoking. In addition, SES was included because we were interested in examining differences in tax pass-through by SES. Age (AKA “age”) was represented by four variables: proportion of people aged 10–19, 20–39, 40–64, 65 and over [31]. Race was simplified to proportion of non-Hispanic white (AKA “race”) where a lower proportion means higher proportion of non-white or Hispanic. A socio-economic composite index (AKA “SES”) was derived as others have done [32] using: log per capita income; log median owner value; proportion of residents: with income from interest/dividends/rent, with high-school education, with bachelor’s degree, and with managerial employment. SES variables were normalized, averaged, and then converted into a percentile according to the normal distribution where 0% and 100% represents neighborhoods with the lowest and highest SES, respectively. 

#### 2.1.7. Region and Urbanicity

Region was defined following census categories: Northeast, Midwest, South and West [33]. Urbanicity was based on county population size: large metro area of 1+ million residents, small metro area of less than 1 million residents, micropolitan urban areas (centered on an urban area with population 10,000 to 49,999), and non-core (all other areas smaller than micropolitan) [34].

#### 2.1.8. Additional Variables: Store Type and State Funding for Tobacco Control Programs

Additional variables were used as control variables in analyses due to their potential to confound the tax-price association. Type of retailer (AKA “store type”), defined as chain supermarket vs. drug store, came from the IRI dataset. Total state tobacco control appropriations 2001–2011 (AKA “tobacco control funding”) came from University of Illinois Chicago’s Bridging the Gap/ImpacTeen Project [35]. They compiled data on public funds allocated by each state for tobacco prevention and control. The allocated funds originate from federal and state sources as well as foundation grants given to states (American Legacy Foundation and Robert Wood Johnson Foundation). Tobacco control funding was included in our analyses to proxy population norms regarding smoking [36]. We converted to dollars per capita (per state population) and adjusted for inflation. 

### 2.2. Statistical Methods

We used a generalized linear model (appropriate for normally distributed outcomes) with random intercepts that accounted for clustering by state and county (appropriate for geographically nested data). Time, region, urbanicity, area-level age distributions, state tobacco control funding, store type, were first added to the model to assess variation in price accounted for by temporal and geographical attributes; then variables of interest were added (state taxes, and bans); socio-demographic variables (SES and race); and then interactions between state taxes and bans.

The model looks as follows:

(1) $$\begin{array}{cc} Cigarette\;Price_{i} & =\;\beta_{0}+u_{jk}+u_{j}+\beta_{1\ldots 10}Year_{1..10}+\beta_{11,\;12,\;13}Region_{NE,\;S,\;MW}\\ & +\beta_{14,15}Urbanicity_{rural,\;small\;metro}+\beta_{16}Cigarettte\;Tax_{j}\\ & +\beta_{17}Hospitality\;Smoking\;Ban_{jk}+\;\beta_{18}SES_{i}+\;\beta_{19}Race_{i}\\ & +\;\beta_{20}Cigarettte\;Tax_{j}\;\times \;Hospitality\;Smoking\;Ban_{jk}+\;\ldots +\;e_{jki} \end{array}$$

where *j* is a state, *k* is a county within that state, and *i* is a store within that county. Note that “…“ includes four parameters for age, one for tobacco control funding, and one for store type.

(2) $$\begin{array}{l} Smoking\;Prevalence_{k}\\ \;=\;\beta_{0}+u_{jk}+u_{j}+\beta_{1\ldots 10}Year_{1..10}+\beta_{11,\;12,\;13}Region_{NE,\;S,\;MW}\\ \;+\beta_{14,15}Urbanicity_{rural,\;small\;metro}+\beta_{16}Cigarettte\;Tax_{j}\\ \;+\beta_{17}Hospitality\;Smoking\;Ban_{k}+\beta_{18}Cigarette\;Price_{k}+\;\beta_{19}SES_{k}\\ \;+\;\beta_{20}Race_{k}+\;\beta_{21}Cigarettte\;Tax_{j}\;\times \;Hospitality\;Smoking\;Ban_{jk}\\ \;+\;\ldots +\;e_{jk} \end{array}$$

where *j* is a state and *k* is a county within that state. Note that “…“ includes four parameters for age, and one for tobacco control funing.

In the price model, we included dummy variables for each year rather than a linear time trend in order to account for spurious time correlation between taxes and prices as there are potentially a number of other factors that could have led to changes in the price of cigarettes, most notably the federal tax increase, but also other factors such as operating costs of cigarette vendors, shipping prices and tobacco prices. Price was non-linear over time and a dummy coding approach is consistent with the modeling approach of other studies [5,7,10]. For the smoking prevalence model, smoking was only somewhat non-linear thus, main results are reported using a more parsimonious model that included linear time + quadratic time + dummy variable for federal cigarette tax. 

Variance decomposition was assessed via intraclass correlation coefficients and state- and county- residual plots (following work by others [37]) in order to illustrate variation of cigarette prices and smoking prevalence across the study area before and after accounting for covariates.

Sensitivity analyses used a fixed effects model that dummy-coded state-county instead of using random intercept terms. Results were virtually the same to those reported using the random intercepts; those results are shown in the Supplement (Tables S1 and S2) and not discussed further.

## 3.Results

### 3.1. Descriptives

**Figure 1:**
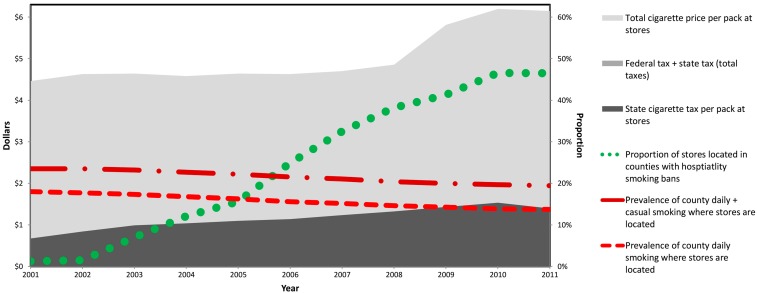
Per pack cigarette price and taxes at chain supermarkets and drug stores, county hospitality indoor smoking bans, and county smoking prevalence, 2001–2011. All prices and taxes were adjusted to 2010 dollars.

Figure 1 shows store-level characteristics for 2001–2011: cigarette prices, taxes and hospitality bans, and smoking prevalence (all prices/taxes were indexed to 2010 dollars). Over the study period, average cigarette PPP rose from $4.46 to $6.15 (a $1.69 or 38% increase). During this time federal taxes rose from $0.43 to $1.01 with the largest increase in April 2009 [38]. Minus federal- and state tobacco taxes, store-level prices rose from $3.80 to $4.77 and accounted for most of the total price of a pack, although on average it represented a slightly declining share of the price (decreased from 85% to 78% of the total PPP). The percent of stores in states-counties with hospitality smoking bans rose from 1% to 46%. Total smoking prevalence declined from 23.5% to 19.4% (4.1 points or 17% lower) but the decline was only evident in the prevalence of daily smokers. (By 2011, counties included in our dataset matched the prevalence of current smoking in the U.S. as a whole: 20% [39]). 

Table 1 shows descriptive data for store-level cigarette price, policy exposures and smoking rates stratified by region, urban class and then by area-level attributes. Averaged across years, stores in the West and Northeast had the highest cigarette prices. Stores in less urbanized areas and in the southern U.S. had the lowest cigarette prices and state taxes accounted for a much lower proportion of the total price, state/county hospitality bans were less prevalent, and daily smoking more prevalent. For example, in the south: state taxes were only 11% of the price of a pack, only 5% of stores were in areas that had hospitality bans, and 17.1% of adult residents were daily smokers. In areas with higher SES, cigarette prices were higher, daily smoking was lower, and state/county bans more prevalent (by the end of the study period).

### 3.2. Regression Models

#### 3.2.1. Price Outcome

After adjustment, the magnitude of cigarette price differences by region and urbanicity was diminished, but was still apparent (Table 2, adjusted for year, cigarette taxes, state/county smoking bans, and age distribution, state tobacco control funding, store type, SES, and proportion non-Hispanic white). Western areas had the most expensive cigarette prices and Midwest and South the cheapest: relative to the West, per-pack prices in these regions were $0.42 and $0.28 cheaper, respectively (*p* < 0.0001, Table 2 model 1.4). In adjusted models, micropolitan and non-core areas had the lowest cigarette prices than large metros ($0.15 and $0.19 cheaper, respectively).

State excise taxes were only partially passed onto the consumer and the presence of bans was associated with a slightly higher PPP. After adjusting for year (including federal tax), region, urbanicity, state/county smoking bans, and age distribution, state tobacco control funding, store type, SES, and proportion non-Hispanic white, a $1 increase in state tax was associated with $0.90 increase in PPP and the presence of bans was associated with a $0.09 higher PPP, *p* < 0.0001 (Table 2 Model 1.4). In the presence of bans the state tax pass-through was slightly higher (0.852 + 0.096 = 0.95, *p* for interaction >0.0001). Price of cigarettes was higher with neighborhood SES index ($0.02, per 10% increase in SES index) but was not associated with proportion non-Hispanic white and there was no evidence of an interaction between state tax and either SES or race (*p* > 0.2).

Supplement Figure S1A illustrates state and store-level differences in prices. Table 2 variance decomposition analyses confirmed that full adjustment for taxes, bans, and area-level characteristics accounted for much of this variability: before and after full adjustment intraclass correlation decreased from 30% to 15%.

#### 3.2.2. Smoking Outcome

Midwest, Northeast and Southern regions had daily smoking prevalence at least 2 percentage points higher than the Western region (Table 1). Supplement Figure S1B, illustrates that relative to other states, daily smoking was lowest in Utah and California and highest in Oklahoma, Tennessee and West Virginia. 

Comparing adjusted models for daily and non-daily smoking, state tobacco tax appeared to have a stronger inverse association with daily smoking. Hospitality bans had an inverse association with both daily and non-daily smoking (Table 3). For example, at an equivalent level of cigarette taxes and prices, the presence of bans was associated with −0.12 and −0.18 percentage point reduction in non-daily and daily smoking, respectively. Interaction results suggest that the presence of bans strengthened the inverse association between cigarette taxes and daily smoking but did not impact the association for non-daily smoking (interaction *p* < 0.0001 and *p* = 0.9, respectively). For example, for each $1 increase in cigarette tax, the expected prevalence of daily smokers decreased by −0.28 percentage points in areas without smoking bans and by −0.53 percentage points in areas with bans (−0.282 − 0.247 = −0.53), after adjusting for geographic factors and covariates (Statistical note: in this calculation, the main effect of ban is omitted because it does not describe the effect of changing taxes on price).

## 4. Discussion

### 4.1. Main Finding of This Study

Using a large dataset of chain supermarkets and drug stores between 2001 and 2011, we found wide variation in store-level cigarette prices that was in part due to differences in state taxes. Cigarette excise taxes were only partially passed onto consumers (a $1 tax was associated with a $0.9 increase in PPP) and pass-through rates did not differ based on area-level socio-demographics but was slightly higher in areas with hospitality bans. Hospitality bans were associated with a slight increase in cigarette price ($0.17 PPP adjusted for state tax, year, area-level age, SES index, and race). Exploratory work analyzing county-level daily smoking found higher taxes and bans inversely associated with daily smoking and impacts were stronger in the presence of the other (indicating a synergistic association). 

### 4.2. What Is Already Known on This Subject and What This Study Adds

Smoking prevalence has declined over time [1,40] yet remains a significant public health problem and varies by locale. One potential reason for local heterogeneity is due to differences in how much tobacco taxes are passed onto consumers. Our finding, that taxes were only partially passed on, was consistent with two studies that used disaggregated store-level UPC-level data. Harding (2012) used a shorter time period and found a pass-through of approximately 0.85 among a household panel and Chiou (2014) used data from supermarkets in a single municipality (the Chicago area) and found an average pass-through of 0.80 [5,28]. Other studies that reported taxes were fully passed or over-shifted onto consumers while using data from supermarkets only or fewer municipalities or price data from individual self-reports [4,8,9,10] Advantages of the dataset we used over other studies were: use of prices of specific goods rather than average price paid which may reflect substitution; included many more years of data relative [4,7,8] and offered better spatial resolution [9,10]. Our findings suggest that cigarette price varied by SES but the variation was small and in general, state cigarette taxes were equitably passed to consumers as there was no evidence that associations between state excise tax and price were different based on area-level SES or minority composition of residents near the chain stores. However, more work on this topic is needed as Harding (2012), using 2 years of household panel data, found that the pass though was less than full throughout the sample but increased with panelists’ household income (with increases in income, pass through rose from 0.811 to 0.897).

Prior studies have estimated that, in the U.S., a 10% increase in price would reduce per capita tobacco consumption between 1% and 6% (price elasticities ranging from −0.1 to −0.6) [2,41,42]. Our exploratory analyses confirmed that after adjusting for area-level characteristics and temporal trend and tobacco prices independent of state tax, a $1 increase in the state cigarette tax was associated with average 0.44 lower daily smoking prevalence (−0.44, *p* < 0.0001), although there was minimal effect on non-daily smoking. If we assume that 90 cents out of $1 state cigarette tax is shifted to consumers, average price elasticity is −0.1 (or if fixed to 2011 prices and daily smoking prevalence it would be −0.2), thus within the range found in prior literature. Reasons that our estimates are slightly lower are: our data are from the 2000s and elasticity estimates were strongest in the U.S. in the 1990s [42]; most studies did not use prices from stores located in a large area that was primarily urbanized. In addition, most studies did not distinguish between daily and non-daily smoking but they benefited from having individual-level data whereas we only reported county-level estimates. 

Our results may suggest that in the presence of smoking bans, state cigarette taxes could be more effective in reducing daily smoking (the presence of hospitality bans strengthened the inverse association between cigarette taxes and daily smoking prevalence, see Section 3.2.2). The effect of hospitality bans itself on smoking prevalence was quite small although statistically significant for county-level non-daily smokers who are sometimes referred to as “social smokers” [43,44] (as opposed to daily smokers). As hospitality bans become more prevalent, more research will be needed on their effects across diverse geographies and on diverse smoking outcomes measured at the individual level.

On average, state taxes were <25% of the total price of cigarettes and <50% of counties in this study had hospitality bans by the end of the study period. Our data primarily represented urbanized areas nevertheless results suggested that policies varied regionally and by urbanicity, with counties in less urbanized areas and in the southern United States having relatively low state taxes and low prevalence of county hospitality bans. Variations in regulations have been shown to give rise to tax avoidance via internet purchases and purchases from bordering states with lower tax rates [5,8,45,46], limiting the efficacy of state-level cigarette taxes. A more effective strategy might be to implement a higher federal cigarette tax rate, as a federal tax would apply uniformly across the United States.

### 4.3. Limitations

This study used a very detailed novel price dataset that included many items, specified brand, and prices were temporally and spatially resolved. However, the dataset only included chain grocery and drug store prices; and stores were almost exclusively in urbanized areas (and excluded New York City and Chicago stores, see methods) thus, generalizability of prices are limited to these venues/contexts. Cigarette carton price was not included thus generalizability of results is limited to cigarette packs. Some work has found higher pass through for cartons [28] while others have found lower pass through for cartons [8,11]. We carefully constructed an average price per store using methods developed by others [7] and that minimized biases specific to our research question (see details in Section 2.1.5); nevertheless, we note that other aggregation methods may generate different results. We did not control or stratify for discount cigarettes (where pass through may be higher [28], see methods for rationale) or store proximity to a jurisdiction with low-taxes/no ban (where pass through may be lower [28] and may have generated non-differential measurement error in the smoking analyses may have weakened the estimates). We were unable to include bans enacted by cities within state-counties that did not have any ban. This omission would not have impacted county-level analyses (Table 3 smoking outcome). Note that among the bans enacted in our study area and time period, bans were commonly enacted at the state level (61% of states in our IRI database enacted ordinances prior to or coincident with our study period) thus obviating the need to assess city ordinances [24]. Nevertheless, possible that this omission could have impacted ban estimates on price outcomes (Table 2 price outcome). Finally, this study did not have access to individual-level data on purchases and smoking status nor number of cigarettes smoked per day and number of quit attempts, smoking outcomes that others have found are responsive to price increases [47,48,49]. Despite its limitations, our study makes a clear contribution to the literature due to its ability to characterize local variation, adjust for local context, and include a long study period and large geographic coverage.

## 5. Conclusions

Cigarette prices at chain stores varied widely within and between states and counties and were only partially explained by differences in state taxes. Wide variation may weaken the effectiveness of these policies at reducing smoking prevalence. Consistent rigorous policies across county and state borders—such as higher federal taxes and/or a federal hospitality smoking ban—may be needed in order to further reduce average smoking prevalence. 

## Figures and Tables

**Table 1:** Characteristics of stores in the dataset: cigarette prices (per pack), taxes (per pack), hospitality bans, and smoking prevalence by region, urbanicity, and area-level socio-demographic characteristics.

Variable	Number of Stores	Cigarette Prices		State Cigarette Taxes		Hospitality Smoking Bans		Daily Smoking Prevalence		Casual Smoking Prevalence	
		Mean	STD	Mean	STD	Mean %	STD	Mean %	STD	Mean %	STD
Overall (2001–2011)	2973	$4.96	$0.90	$1.06	$0.69	21.4	34.2	16.1	3.9	5.8	0.8
*Region*											
Northeast	707	$5.64	$0.91	$1.81	$0.57	43.9	41.1	16.1	3.2	5.6	0.6
Midwest	547	$4.70	$0.64	$1.01	$0.54	21.4	29.9	18.2	3.6	5.9	0.8
South	1032	$4.38	$0.68	$0.50	$0.44	5.0	18.0	17.1	4.0	6.0	0.8
West	687	$5.35	$0.70	$1.17	$0.40	22.8	34.7	13.0	2.7	5.6	0.8
*Urban classification (county level variable)*											
Large metro (pop. ≥ 1 million)	1948	$5.11	$0.90	$1.16	$0.68	23.5	35.5	14.8	3.5	5.8	0.8
Small metro (pop. < 1 million)	859	$4.72	$0.86	$0.90	$0.69	17.7	31.4	18.3	3.3	5.8	0.7
Micropolitan (pop. 10 k–50 k)	128	$4.54	$0.72	$0.82	$0.57	15.3	30.3	20.3	3.0	5.9	1.0
Noncore areas (pop < 10 k)	38	$4.33	$0.75	$0.66	$0.66	14.6	32.5	21.0	2.8	5.9	0.9
Area-level socio-economic index *											
lowest tertile (0.2–<35)	991	$4.73	$0.85	$0.92	$0.66	15.4	30.4	16.7	4.1	5.7	0.8
middle tertile (35–<62)	991	$5.03	$0.90	$1.10	$0.70	22.5	34.5	16.6	3.9	5.8	0.8
highest tertile (62–100)	991	$5.14	$0.92	$1.17	$0.70	26.3	36.6	15.1	3.5	5.8	0.8
Area-level non-Hispanic white *^,^^**†**^											
lowest tertile (2.6%–<72%)	991	$4.88	$0.89	$0.92	$0.66	15.9	31.9	18.0	4.0	6.0	0.8
middle tertile (72%–<87%)	991	$4.98	$0.91	$1.04	$0.69	20.9	33.6	16.3	3.6	5.7	0.8
highest tertile (87%–100%)	991	$5.04	$0.90	$1.22	$0.70	27.3	36.0	14.1	3.2	5.7	0.8

* Area-level refers to block group cluster; ^**†**^ Hispanic and non-white were collapsed in order to obtain sufficient population numbers across block groups. “non-Hispanic White” can also be interpreted as percent that are Hispanic or non-white (1-non-Hispanic white), thus the tertile distribution is 29%–97%, 13.4%–29%, %0–13.4%.

**Table 2:** Mean differences in store-level cigarette prices per pack; data from 2001 to 2011, *n* = 2973 chain supermarkets and convenience stores; estimates are derived from a random intercept model *.

Explanatory Variable	Labels and Category	Model 1.1 Base Adjustment ^†^			Model 1.2 Base ^†^ Model Plus State Tax				Model 1.3 Base ^†^ Model Plus Hospitality Smoking Ban		Model 1.4 Base ^†^ Model Plus State Tax, Hospitality Smoking Bans, SES Index, Race		Model 1.5 Base ^†^ Model Plus State Tax, Hospitality Smoking Bans, SES Index, Race, Interaction Tax × Ban	
		Est.	*p*-Value		Est.		*p*-Value		Est.	*p*-Value	Est.	*p*-Value	Est.	*p*-Value
**Region**	North East	0.290	0.1481		−0.307		0.0007		0.226	0.2176	−0.302	0.0006	−0.302	0.0006
	Midwest	−0.634	0.0013		−0.426		<0.0001		−0.612	0.0007	−0.419	<0.0001	−0.418	<0.0001
	South	−0.835	<0.0001		−0.304		0.0003		−0.770	<0.0001	−0.279	0.0007	−0.304	0.0002
	West	Ref			Ref				Ref		Ref		Ref	
**Urbanicity ^‡^**	Small metro	−0.109	0.0011		−0.096		0.0024		−0.110	0.0009	−0.081	0.0106	−0.080	0.0108
	Micropolitan	−0.220	<0.0001		−0.189		<0.0001		−0.216	<0.0001	−0.148	0.0017	−0.150	0.0015
	Noncore	−0.240	0.0008		−0.244		0.0004		−0.241	0.0008	−0.191	0.0053	−0.195	0.0043
	Large metro	Ref			Ref				Ref		Ref		Ref	
**State tax**	State cigarette tax, per pack				0.912		<0.0001				0.895	<0.0001	0.852	<0.0001
**Ban**	Hospitality (restaurant and bar) indoor smoking ban								0.313	<0.0001	0.089	<0.0001	−0.070	0.0650
**Interaction**	Tax × Ban												0.096	<0.0001
**Area-level ^§^**	Socio-economic index										0.002	<0.0001	0.002	<0.0001
	Race non-Hispanic white										0.000	0.7	0.000	0.7
Intraclass correlation coefficients *														
	Both state and county	0.30			0.16				0.27		0.15		0.15	

Est. = Estimate. Ref = Referent value; * Random intercepts were included for state and county. Intraclass correlation is the proportion of variation in price that is accounted for by price differences at the state and county levels; **^†^** Base adjustment. Time was a dummy variable in the model (year 2001–2011) and accounts for federal cigarette tax. Additional base adjustment variables are: region, urbanicity, state tobacco control funding, store type, and area-level age (percent of population aged 10–19, 20–39, 40–64, 65+); **^‡^** Urbanicity is a county-level variable (see methods); **^§^** Area-level refers to block group cluster. Area-level socio-economic index units are displayed in 10 percentile increments (cigarette price increases $0.02 per 10% increase in socio-economic index).

**Table 3:** Adjusted mean difference in county-level smoking prevalence according to cigarette price, state tax, hospitality ban and interactions, data from 2001 to 2011, *n* = 477 U.S. counties. Estimates are derived from a random intercept model *.

Explanatory Variable	Label of Variable	Model 2.1 Base Adjustment ^†^		Model 2.2 Base ^†^ Adjustment, Cigarette Price, State Tax, Hospitality Bans		Model 2.3 Base ^†^ Adjustment, SES Index, Race, Cigarette Price, State Tax, Hospitality Bans, Interaction Tax × Bans	
		Est.	*p*-Value	Est.	*p*-Value	Est.	*p*-Value
**A. Daily smoking prevalence**							
County cigarette price (after adjustment for taxes)		0.010	0.621	0.022	0.269	0.015	0.46
State tax	State cigarette tax, per pack	−0.433	<0.001	−0.440	<0.001	−0.282	<0.001
Ban	Hospitality (restaurant and bar) indoor smoking ban			−0.123	0.003	0.311	<0.001
Interaction	Ban × state tax					−0.247	<0.001
**B. Non-daily (casual) smoking prevalence**							
County cigarette price (after adjustment for taxes)		0.039	0.002	0.041	0.001	0.041	0.001
State tax	State cigarette tax, per pack	−0.124	<0.001	−0.096	<0.001	−0.092	<0.001
Ban	Hospitality (restaurant and bar) indoor smoking ban			−0.182	<0.001	−0.18	0.001
Interaction	Ban × state tax					−0.003	0.918

Est. = Estimate. Ref = Referent value; * Random intercepts were included for counties (within-states); **^†^** Base adjustment. All models include time which was entered as a linear term for year + year squared + dummy variable to indicate before or after year 2009 (the year when the federal tax increased across all U.S. states). Additional base adjustment variables are: region, urbanicity, cigarette price, state tobacco control funding, store type, and area-level age (percent of population aged 10–19, 20–39, 40–64, 65+).

## Supplementary Materials

The following are available online at www.mdpi.com/1660-4601/14/3/318/s1, Figure S1: Variation by state in (A) average cigarette price per pack by state (B) daily smoking prevalence; with 95% confidence intervals. Y-axis is the mean residual and X-axis displays rankings low to high. Y-axis residuals means that 0 is the overall average, −1.0 on the y-axis means the value is −1.0 than average, Table S1: Sensitivity to using fixed effects model. Mean differences in store-level cigarette prices per pack; data from 2001 to 2011, *n* = 2973 chain supermarkets and convenience stores, Table S2: Sensitivity to using fixed effects model. Adjusted mean difference in county-level smoking prevalence according to cigarette price, state tax, hospitality ban and interactions, data from 2001 to 2011.
