# Supplementary material for: Exploring Impacts of Taxes and Hospitality Bans on Cigarette Prices and Smoking Prevalence Using a Large Dataset of Cigarette Prices at Stores 2001–2011, USA
Source: Int J Environ Res Public Health. 2017 Mar 20;14(3):318. doi: 10.3390/ijerph14030318 (PMC5369154; doi:10.3390/ijerph14030318)
Supplement: Supplementary file 1 [file ijerph-14-00318-s001.pdf]

# Supplementary Materials: Exploring Impacts of Taxes and Hospitality Bans on Cigarette Prices and Smoking Prevalence Using a Large Dataset of Cigarette Prices at Stores 2001–2011, USA

**Lance S. Ballester, Amy H. Auchincloss, Lucy F. Robinson and Stephanie L. Mayne**

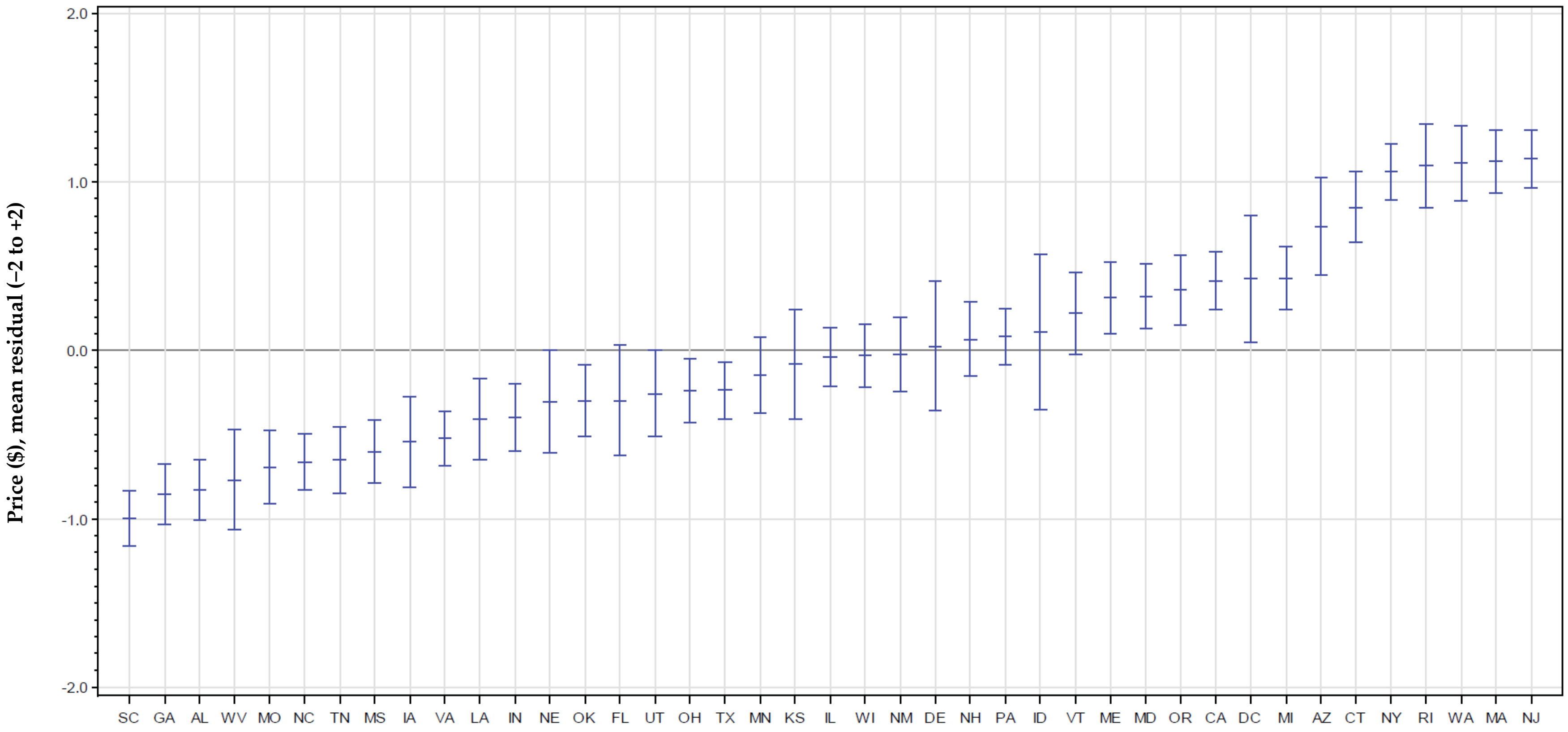


**Unadjusted (except for county random intercept)**
**(A)**

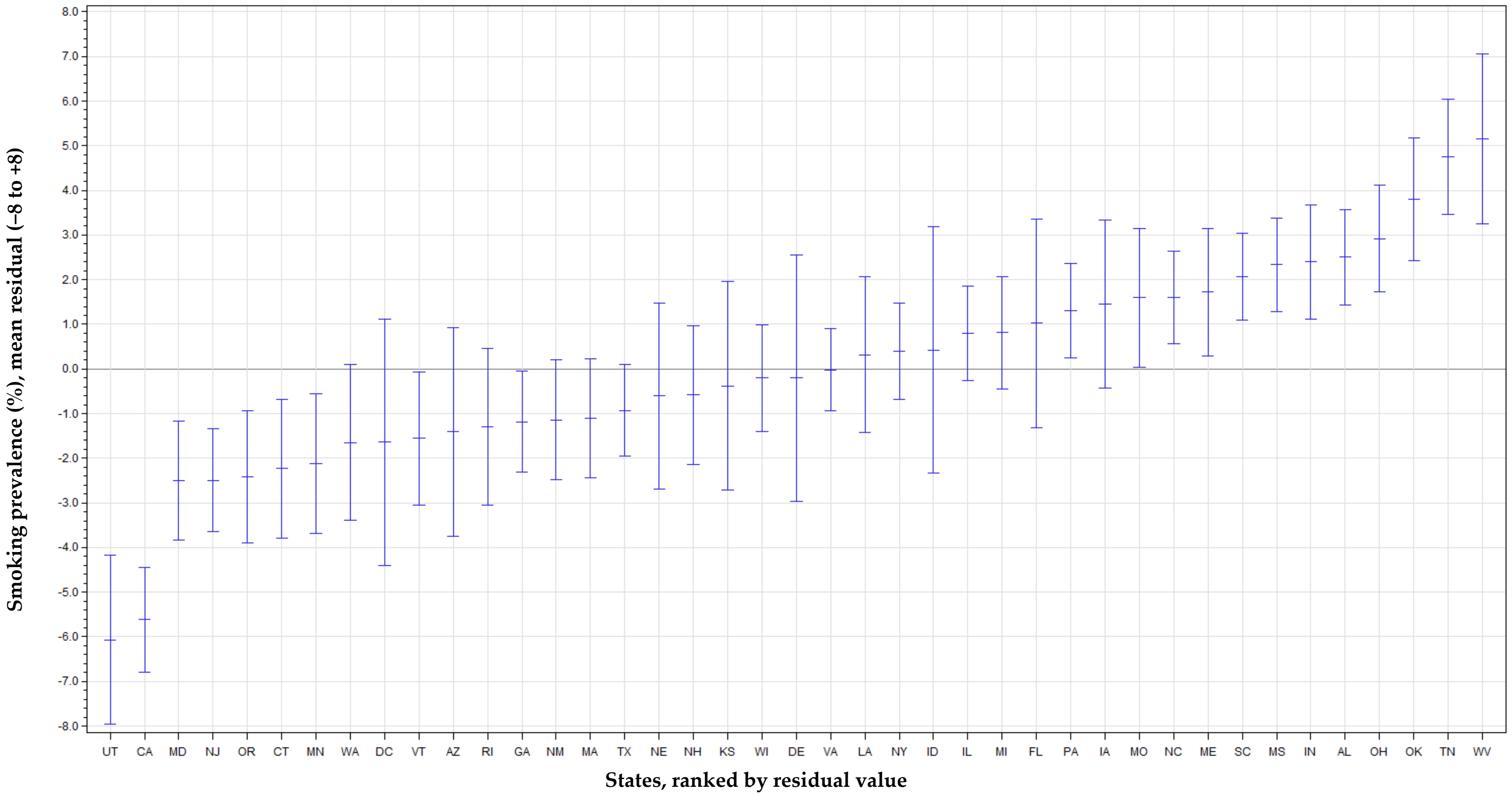


(**B**)

**Figure S1.** Variation by state in (**A**) average cigarette price per pack by state (**B**) daily smoking prevalence; with 95% confidence intervals. Y-axis is the mean residual and X-axis displays rankings low to high. Y-axis residuals means that 0 is the overall average, −1.0 on the y-axis means the value is −1.0 than average.

**Table S1. Sensitivity to using fixed effects**. Mean differences in store-level cigarette prices per pack; data from 2001–2011, *n* = 2973 chain supermarkets and convenience stores; estimates are derived from a fixed effects model *.

| Variable | Label | Model 1.2 Base Model † Plus State Tax | | Model 1.3 Base Model † Plus Hospitality Smoking Ban | | Model 1.4 Base Model † Plus State Tax, Hospitality Smoking Bans, SES Index, Race | | Model 1.5 Base Model † Plus State Tax, Hospitality Smoking Bans, SES Index, Race, Interaction Tax × Ban | |
|---|---|---|---|---|---|---|---|---|---|
| | | Est | *p*-Value | Est | *p*-Value | Est | *p*-Value | Est | *p*-Value |
| **State tax** | State cigarette tax, per pack | 0.920 | <0.0001 | | | 0.904 | <0.0001 | 0.852 | <0.0001 |
| **Ban** | Hospitality (restaurant and bar) indoor smoking ban | | | 0.313 | <0.0001 | 0.086 | <0.0001 | −0.077 | 0.0477 |
| **Interaction** | Tax × Ban | | | | | | | 0.096 | <0.0001 |
| **Area-level** ‡ | Socio-economic index | | | | | 0.001 | <0.0001 | 0.002 | <0.0001 |
| | Race non-Hispanic white | | | | | 0.000 | <0.0001 | 0.000 | 0.2701 |

Est = Estimate. Ref = Referent value; * Dummy variables were included for state-county; † Base adjustment. All models include dummy variables for time (year 2001–2011); note that year also accounts for federal cigarette tax. Additional covariates: state tobacco control funding, area-level age (percent of population aged 10–19, 20–39, 40–64, 65+). For each store, region, urbanicity, and store type do not change over time thus are not utilized in the fixed effects model; ‡ Area-level refers to block group cluster. Area-level socio-economic index units are displayed in 10 percentile increments (cigarette price increases $0.02 per 10% increase in socio-economic index).

**Table S2. Sensitivity to using fixed effects**. Adjusted mean difference in county-level smoking prevalence according to cigarette price, state tax, hospitality ban and interactions, data from 2001–2011. Estimates are derived from a fixed effects model *.

| Variable | Label | Model 2.1 Base Adjustment †, Cigarette Price, State Tax | | Model 2.2 Base Adjustment †, Cigarette Price, State Tax, Hospitality Bans | | Model 2.3 Base Adjustment †, SES Index, Race, Cigarette Price, State Tax, Hospitality Bans, Interaction Tax × Bans | |
|---|---|---|---|---|---|---|---|
| | | Estimate | *p*-Value | Estimate | *p*-Value | Estimate | *p*-Value |
| A. Daily smoking prevalence | | | | | | | |
| County cigarette price (after adjustment for taxes) | | 0.022 | 0.279 | 0.023 | 0.251 | 0.026 | 0.187 |
| State tax | State cigarette tax, per pack | −0.459 | <0.0001 | −0.441 | <0.0001 | −0.316 | <0.0001 |
| Ban | Hospitality (restaurant and bar) indoor smoking ban | | | −0.122 | 0.003 | 0.274 | 0.001 |
| Interaction | Ban × state tax | | | | | −0.249 | <0.0001 |
| B. Non-daily (casual) smoking prevalence | | | | | | | |
| County cigarette price (after adjustment for taxes) | | 0.04 | 0.002 | 0.042 | 0.001 | 0.042 | 0.001 |
| State tax | State cigarette tax, per pack | −0.127 | <0.0001 | −0.1 | <0.001 | −0.1 | 0.001 |
| Ban | Hospitality (restaurant and bar) indoor smoking ban | | | −0.177 | <0.0001 | −0.176 | 0.001 |
| Interaction | Ban × state tax | | | | | −0.001 | 0.981 |

Est = Estimate. Ref = Referent value; * Dummy variables were included for state-county; † Base adjustment. All models include time which was entered as a linear term for year + year squared + dummy variable to indicate before or after year 2009 (the year when the federal tax increased across all U.S. states); cigarette price; state tobacco control funding; and area-level age (percent of population aged 10–19, 20–39, 40–64, 65+). For each store, region and urbanicity do not change over time thus are not utilized in the model.

© 2017 by the authors; licensee MDPI, Basel, Switzerland. This article is an open access article distributed under the terms and conditions of the Creative Commons by Attribution (CC-BY) license (http://creativecommons.org/licenses/by/4.0/).
